# 3-(4-Chloro­phen­yl)-1-[(2*R*,4a*R*,8a*R*)-4a,8-dimethyl-1,2,3,4,4a,5,6,8a-octa­hydro­naphthalen-2-yl]prop-2-en-1-one

**DOI:** 10.1107/S1600536811016886

**Published:** 2011-05-07

**Authors:** Mohamed Tebbaa, Ahmed Benharref, Moha Berraho, Daniel Avignant, Abdelghani Oudahmane, Mohamed Akssira

**Affiliations:** a Laboratoire de Chimie Bioorganique et Analytique, URAC 22 BP 146, FSTM, Université Hassan II, Mohammedia-Casablanca 20810 Mohammedia, Morocco; b Laboratoire de Chimie Biomoleculaire, Substances Naturelles et Réactivité, URAC16, Université Cadi Ayyad, Faculté des Sciences Semlalia, BP 2390, Bd My Abdellah, 40000 Marrakech, Morocco; c Université Blaise Pascal, Laboratoire des Matériaux Inorganiques, UMR CNRS 6002, 24 Avenue des Landais, 63177 Aubière, France

## Abstract

The title compound, C_21_H_25_ClO, was semi-synthesized from isocostic acid, isolated from the aerial part of *Inula Viscosa­* (L) Aiton [or *Dittrichia Viscosa­* (L) Greuter]. The cyclo­hexene ring has a half-chair conformation, whereas the cyclo­hexane ring displays a chair conformation.

## Related literature

For background to the medicinal inter­est in *Inula Viscosa­* (L) Aiton [or *Dittrichia Viscosa­* (L) Greuter], see: Shtacher & Kasshman (1970 ▶); Bohlman & Gupta (1982 ▶); Azoulay *et al.* (1986 ▶); Bohlman *et al.* (1977 ▶); Ceccherelli *et al.* (1988 ▶). For the synthesis, see: Kutney & Singh (1984 ▶). For conformational analysis, see: Cremer & Pople (1975 ▶).
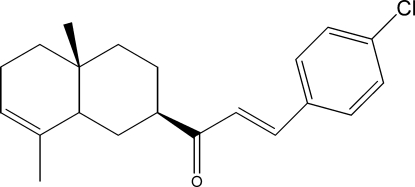


## Experimental

### 

#### Crystal data

C_21_H_25_ClO
*M* *_r_* = 328.86
Monoclinic, 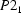

*a* = 10.9869 (5) Å
*b* = 7.0054 (3) Å
*c* = 12.1883 (6) Å
β = 105.726 (2)°
*V* = 902.99 (7) Å^3^
*Z* = 2
Mo *K*α radiation
μ = 0.21 mm^−1^
*T* = 298 K
0.28 × 0.20 × 0.16 mm

#### Data collection

Bruker X8 APEX CCD area-detector diffractometer
6963 measured reflections
3358 independent reflections
2713 reflections with *I* > 2σ(*I*)
*R*_int_ = 0.021

#### Refinement

*R*[*F* ^2^ > 2σ(*F* ^2^)] = 0.039
*wR*(*F* ^2^) = 0.106
*S* = 1.08
3358 reflections
210 parameters
1 restraint
H-atom parameters constrained
Δρ_max_ = 0.18 e Å^−3^
Δρ_min_ = −0.21 e Å^−3^
Absolute structure: Flack (1983 ▶), 1373 Friedel pairs
Flack parameter: −0.11 (7)

### 

Data collection: *APEX2* (Bruker, 2005 ▶); cell refinement: *SAINT* (Bruker, 2005 ▶); data reduction: *SAINT*; program(s) used to solve structure: *SHELXS97* (Sheldrick, 2008 ▶); program(s) used to refine structure: *SHELXL97* (Sheldrick, 2008 ▶); molecular graphics: *ORTEP-3 for Windows* (Farrugia, 1997) ▶; software used to prepare material for publication: *WinGX* (Farrugia, 1999 ▶).

## Supplementary Material

Crystal structure: contains datablocks I, global. DOI: 10.1107/S1600536811016886/ci5186sup1.cif

Structure factors: contains datablocks I. DOI: 10.1107/S1600536811016886/ci5186Isup2.hkl

Supplementary material file. DOI: 10.1107/S1600536811016886/ci5186Isup3.cml

Additional supplementary materials:  crystallographic information; 3D view; checkCIF report

## Figures and Tables

**Fig. 1.**
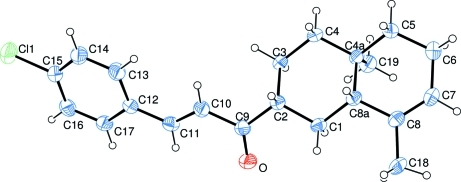
Molecular structure of the title compound with the atom-labelling scheme. Displacement ellipsoids are drawn at the 30% probability level. H atoms are shown as small spheres of arbitrary radii.

## supplementary crystallographic information

### Comment

Isocostic acid is the main constituent of the dichloromethane extract of the aerial part of Inula viscosa (*L*) Aiton or Dittrichia Viscosa (*L*) Greuter. This plant is widespread in Mediterranean area and extends to the Atlantic cost of Morocco. It is a well known medicinal plant (Shtacher & Kasshman, 1970; Bohlman & Gupta, 1982) and has some pharmacological activities (Azoulay *et al.*, 1986). This plant has undergone a chemical study in order to isolate sesquiterpene lactones (Bohlman *et al.*, 1977) and sesquiterpene acids (Ceccherelli *et al.*, 1988). The literature does not report any article on the transformation of this acid. In order to prepare products with high added value, we studied the reactivity of this acid. Thus, from this acid, we have prepared by reaction of Curtius the 1-(4a,8-dimethyl-1,2,3,4,4a,5,6,8a-octahydronaphthalen-2-yl)-ethanone which was synthesized by Kutney *et al.*(1984). The condensation of this sesquiterpene ketone with *p*-chlorbenzaldehyde in the presence of sodium hydroxide allows us to obtain the 3-(4-chlorophenyl)-1-(4a,8-dimethyl- 1,2,3,4,4a,5,6, 8a-octahydro-naphthalen-2-yl)prop-2-en-1-one with a good yield of 90%. The structure of this new derivative of isocostic acid was established by NMR spectral analysis of 1H, 13 C and mass spectroscopy and confirmed by its single-crystal X-ray structure.

In the title molecule (Fig. 1), the cyclohexane ring adopts a chair conformation, as indicated by the total puckering amplitude Q(T) = 0.574 (2) Å and spherical polar angle θ = 180.0 (2)° with φ = 48 (13)°. The cyclohexene ring has a half-chair conformation with QT = 0.525 (2) Å, θ =129.8 (2)°, φ = 191.1 (3)° (Cremer & Pople, 1975). Owing to the presence of Cl atom, the absolute configuration could be fully confirmed, by refining the Flack (1983) parameter as C2(*R*), C4a(*R*) and C8a(*R*).

### Experimental

*p*-Chlorobenzaldehyde dissolved in ethanol (20 ml) was added drop by drop to a mixture of 1-(4a,8-dimethyl-1,2,3,4,4a,5,6,8a-octahydronaphthalen- 2-yl)ethanone (1 g, 4.84 mmol), anhydrous ethanol (40 ml), and 2 ml of a solution of sodium hydroxide (2 N, 667 mg, 4.84 mmol). The mixture was stirred for 3 h at room temperature. After neutralization, it was extracted three times with 40 ml of dichloromethane, the organic phase was dried over sodium sulfate and then evaporated under vacuum. The residue was subjected to chromatography on a column of silica gel with hexane-ethyl acetate (97:4) as eluent, to obtain 3-(4-chlorphenyl)-1-(4a, 8-dimethyl-1,2,3,4,4a,5,6,8a- octahydronaphthalen-2-yl)prop-2-en-1-one with a yield of 90%. The title compound was recrystallized in hexane-ethyl acetate (7:3).

### Refinement

All H atoms were positioned geometrically and treated as riding with C-H = 0.93 Å (aromatic), 0.96 Å (methyl), 0.97 Å (methylene), 0.98Å (methine) with *U*_iso_(H) = 1.2U_eq_(C) and 1.5*U*_eq_(C_methyl_).

### Crystal data

**Table:**

C_21_H_25_ClO	*F*(000) = 352
*M**_r_* = 328.86	*D*_x_ = 1.206 Mg m^−^^3^
Monoclinic, *P*2_1_	Mo *K*α radiation, λ = 0.71073 Å
Hall symbol: P 2yb	Cell parameters from 3362 reflections
*a* = 10.9869 (5) Å	θ = 3.5–26.3°
*b* = 7.0054 (3) Å	µ = 0.21 mm^−^^1^
*c* = 12.1883 (6) Å	*T* = 298 K
β = 105.726 (2)°	Prism, colourless
*V* = 902.99 (7) Å^3^	0.28 × 0.20 × 0.16 mm
*Z* = 2	

### Data collection

**Table:**

Bruker X8 APEX CCD area-detector diffractometer	2713 reflections with *I* > 2σ(*I*)
Radiation source: fine-focus sealed tube	*R*_int_ = 0.021
graphite	θ_max_ = 26.3°, θ_min_ = 4.1°
Detector resolution: 8.3333 pixels mm^-1^	*h* = −13→13
ω and φ scans	*k* = −7→8
6963 measured reflections	*l* = −15→14
3358 independent reflections	

### Refinement

**Table:**

Refinement on *F*^2^	Secondary atom site location: difference Fourier map
Least-squares matrix: full	Hydrogen site location: inferred from neighbouring sites
*R*[*F*^2^ > 2σ(*F*^2^)] = 0.039	H-atom parameters constrained
*wR*(*F*^2^) = 0.106	*w* = 1/[σ^2^(*F*_o_^2^) + (0.0579*P*)^2^] where *P* = (*F*_o_^2^ + 2*F*_c_^2^)/3
*S* = 1.08	(Δ/σ)_max_ = 0.001
3358 reflections	Δρ_max_ = 0.18 e Å^−^^3^
210 parameters	Δρ_min_ = −0.21 e Å^−^^3^
1 restraint	Absolute structure: Flack (1983), 1373 Friedel pairs
Primary atom site location: structure-invariant direct methods	Flack parameter: −0.11 (7)

### Special details

**Table:**

Geometry. All s.u.'s (except the s.u. in the dihedral angle between two l.s. planes) are estimated using the full covariance matrix. The cell s.u.'s are taken into account individually in the estimation of s.u.'s in distances, angles and torsion angles; correlations between s.u.'s in cell parameters are only used when they are defined by crystal symmetry. An approximate (isotropic) treatment of cell s.u.'s is used for estimating s.u.'s involving l.s. planes.

### Fractional atomic coordinates and isotropic or equivalent isotropic displacement parameters (Å^2^)

**Table:**

	*x*	*y*	*z*	*U*_iso_*/*U*_eq_	
C1	−0.12078 (19)	0.1716 (3)	0.28143 (17)	0.0460 (5)	
H1A	−0.1945	0.2468	0.2826	0.055*	
H1B	−0.0877	0.1149	0.3562	0.055*	
C2	−0.02074 (18)	0.3009 (3)	0.25507 (16)	0.0436 (5)	
H2	0.0531	0.2209	0.2575	0.052*	
C3	−0.06528 (19)	0.3826 (3)	0.13502 (16)	0.0481 (5)	
H3A	0.0022	0.4573	0.1189	0.058*	
H3B	−0.1368	0.4666	0.1299	0.058*	
C4	−0.1039 (2)	0.2228 (3)	0.04658 (17)	0.0508 (5)	
H4A	−0.0302	0.1463	0.0471	0.061*	
H4B	−0.1347	0.2790	−0.0286	0.061*	
C4A	−0.20679 (16)	0.0933 (3)	0.06975 (15)	0.0413 (4)	
C5	−0.2281 (2)	−0.0790 (3)	−0.01062 (18)	0.0519 (5)	
H5A	−0.2520	−0.0344	−0.0889	0.062*	
H5B	−0.1495	−0.1495	0.0015	0.062*	
C6	−0.3304 (2)	−0.2119 (3)	0.00779 (19)	0.0600 (6)	
H6A	−0.4126	−0.1619	−0.0326	0.072*	
H6B	−0.3210	−0.3363	−0.0239	0.072*	
C7	−0.3245 (2)	−0.2335 (3)	0.13080 (19)	0.0555 (6)	
H7	−0.3772	−0.3241	0.1498	0.067*	
C8	−0.25005 (18)	−0.1339 (3)	0.21527 (17)	0.0466 (5)	
C8A	−0.15912 (16)	0.0137 (3)	0.19227 (16)	0.0407 (4)	
H8A	−0.0811	−0.0559	0.1945	0.049*	
C9	0.02262 (19)	0.4566 (3)	0.34237 (17)	0.0480 (5)	
C10	0.12607 (19)	0.5813 (3)	0.32607 (18)	0.0526 (5)	
H10	0.1705	0.5438	0.2747	0.063*	
C11	0.1570 (2)	0.7435 (3)	0.38210 (17)	0.0502 (5)	
H11	0.1146	0.7733	0.4363	0.060*	
C12	0.25209 (19)	0.8815 (3)	0.36696 (17)	0.0458 (5)	
C13	0.3308 (2)	0.8509 (4)	0.2960 (2)	0.0618 (6)	
H13	0.3262	0.7353	0.2575	0.074*	
C14	0.4147 (2)	0.9871 (4)	0.2817 (2)	0.0669 (7)	
H14	0.4662	0.9643	0.2338	0.080*	
C15	0.4221 (2)	1.1572 (3)	0.33870 (19)	0.0570 (6)	
C16	0.3477 (2)	1.1907 (3)	0.4105 (2)	0.0597 (6)	
H16	0.3543	1.3056	0.4499	0.072*	
C17	0.2636 (2)	1.0548 (3)	0.42400 (18)	0.0540 (5)	
H17	0.2131	1.0790	0.4725	0.065*	
C18	−0.2510 (2)	−0.1670 (4)	0.33701 (18)	0.0668 (6)	
H18A	−0.3116	−0.2645	0.3397	0.100*	
H18B	−0.2736	−0.0508	0.3684	0.100*	
H18C	−0.1685	−0.2069	0.3807	0.100*	
C19	−0.3293 (2)	0.2045 (3)	0.05233 (19)	0.0544 (5)	
H19A	−0.3572	0.2478	−0.0252	0.082*	
H19B	−0.3155	0.3124	0.1027	0.082*	
H19C	−0.3928	0.1234	0.0685	0.082*	
O	−0.02456 (15)	0.4817 (2)	0.42008 (13)	0.0666 (5)	
Cl1	0.52589 (7)	1.33272 (12)	0.31822 (6)	0.0916 (3)	

### Atomic displacement parameters (Å^2^)

**Table:**

	*U*^11^	*U*^22^	*U*^33^	*U*^12^	*U*^13^	*U*^23^
C1	0.0503 (11)	0.0493 (11)	0.0398 (10)	−0.0030 (9)	0.0149 (9)	0.0080 (9)
C2	0.0450 (10)	0.0452 (12)	0.0426 (10)	−0.0024 (8)	0.0150 (9)	−0.0013 (9)
C3	0.0563 (12)	0.0474 (12)	0.0456 (11)	−0.0116 (9)	0.0222 (9)	0.0044 (9)
C4	0.0547 (12)	0.0623 (14)	0.0407 (11)	−0.0130 (10)	0.0217 (10)	−0.0005 (10)
C4A	0.0398 (9)	0.0441 (10)	0.0415 (10)	−0.0028 (8)	0.0135 (8)	0.0041 (9)
C5	0.0533 (12)	0.0609 (13)	0.0450 (11)	−0.0084 (10)	0.0191 (9)	−0.0081 (10)
C6	0.0604 (13)	0.0575 (14)	0.0615 (14)	−0.0153 (11)	0.0156 (11)	−0.0047 (11)
C7	0.0553 (13)	0.0488 (12)	0.0659 (14)	−0.0105 (10)	0.0223 (11)	0.0054 (11)
C8	0.0482 (11)	0.0431 (11)	0.0505 (11)	0.0023 (9)	0.0169 (9)	0.0100 (9)
C8A	0.0385 (9)	0.0427 (11)	0.0416 (10)	0.0035 (8)	0.0122 (8)	0.0063 (8)
C9	0.0547 (12)	0.0482 (11)	0.0429 (11)	−0.0026 (9)	0.0163 (9)	0.0002 (9)
C10	0.0555 (12)	0.0524 (12)	0.0526 (12)	−0.0058 (10)	0.0193 (10)	−0.0099 (10)
C11	0.0567 (12)	0.0539 (13)	0.0418 (11)	0.0009 (10)	0.0163 (10)	−0.0046 (10)
C12	0.0492 (11)	0.0479 (12)	0.0399 (10)	−0.0019 (9)	0.0113 (9)	−0.0046 (9)
C13	0.0684 (14)	0.0576 (13)	0.0655 (14)	−0.0105 (12)	0.0286 (12)	−0.0241 (12)
C14	0.0655 (14)	0.0746 (16)	0.0695 (15)	−0.0161 (13)	0.0337 (12)	−0.0237 (14)
C15	0.0542 (12)	0.0655 (14)	0.0520 (13)	−0.0140 (11)	0.0157 (10)	−0.0074 (11)
C16	0.0713 (15)	0.0500 (13)	0.0571 (13)	−0.0074 (11)	0.0160 (12)	−0.0165 (11)
C17	0.0615 (13)	0.0556 (13)	0.0493 (12)	−0.0027 (11)	0.0225 (10)	−0.0105 (10)
C18	0.0787 (16)	0.0653 (14)	0.0586 (13)	−0.0192 (13)	0.0222 (12)	0.0195 (13)
C19	0.0495 (12)	0.0541 (13)	0.0560 (13)	0.0075 (10)	0.0083 (10)	0.0123 (10)
O	0.0877 (11)	0.0676 (10)	0.0527 (9)	−0.0183 (9)	0.0331 (8)	−0.0118 (8)
Cl1	0.0968 (5)	0.0864 (5)	0.0996 (5)	−0.0428 (4)	0.0405 (4)	−0.0190 (4)

### Geometric parameters (Å, °)

**Table:**

C1—C2	1.524 (2)	C8—C8A	1.516 (3)
C1—C8A	1.527 (3)	C8A—H8A	0.98
C1—H1A	0.97	C9—O	1.209 (2)
C1—H1B	0.97	C9—C10	1.489 (3)
C2—C9	1.508 (3)	C10—C11	1.322 (3)
C2—C3	1.523 (3)	C10—H10	0.93
C2—H2	0.98	C11—C12	1.471 (3)
C3—C4	1.532 (3)	C11—H11	0.93
C3—H3A	0.97	C12—C17	1.388 (3)
C3—H3B	0.97	C12—C13	1.395 (3)
C4—C4A	1.534 (2)	C13—C14	1.370 (3)
C4—H4A	0.97	C13—H13	0.93
C4—H4B	0.97	C14—C15	1.371 (3)
C4A—C19	1.520 (3)	C14—H14	0.93
C4A—C5	1.532 (3)	C15—C16	1.370 (3)
C4A—C8A	1.546 (2)	C15—Cl1	1.740 (2)
C5—C6	1.522 (3)	C16—C17	1.367 (3)
C5—H5A	0.97	C16—H16	0.93
C5—H5B	0.97	C17—H17	0.93
C6—C7	1.491 (3)	C18—H18A	0.96
C6—H6A	0.97	C18—H18B	0.96
C6—H6B	0.97	C18—H18C	0.96
C7—C8	1.326 (3)	C19—H19A	0.96
C7—H7	0.93	C19—H19B	0.96
C8—C18	1.505 (3)	C19—H19C	0.96
			
C2—C1—C8A	110.86 (15)	C18—C8—C8A	117.95 (18)
C2—C1—H1A	109.5	C8—C8A—C1	115.52 (16)
C8A—C1—H1A	109.5	C8—C8A—C4A	110.91 (15)
C2—C1—H1B	109.5	C1—C8A—C4A	112.44 (15)
C8A—C1—H1B	109.5	C8—C8A—H8A	105.7
H1A—C1—H1B	108.1	C1—C8A—H8A	105.7
C9—C2—C3	111.35 (16)	C4A—C8A—H8A	105.7
C9—C2—C1	112.90 (16)	O—C9—C10	121.57 (19)
C3—C2—C1	111.31 (15)	O—C9—C2	122.51 (18)
C9—C2—H2	107.0	C10—C9—C2	115.91 (18)
C3—C2—H2	107.0	C11—C10—C9	122.3 (2)
C1—C2—H2	107.0	C11—C10—H10	118.8
C2—C3—C4	110.94 (16)	C9—C10—H10	118.8
C2—C3—H3A	109.5	C10—C11—C12	126.3 (2)
C4—C3—H3A	109.5	C10—C11—H11	116.8
C2—C3—H3B	109.5	C12—C11—H11	116.8
C4—C3—H3B	109.5	C17—C12—C13	117.22 (19)
H3A—C3—H3B	108.0	C17—C12—C11	118.91 (19)
C3—C4—C4A	112.32 (15)	C13—C12—C11	123.86 (18)
C3—C4—H4A	109.1	C14—C13—C12	121.5 (2)
C4A—C4—H4A	109.1	C14—C13—H13	119.2
C3—C4—H4B	109.1	C12—C13—H13	119.2
C4A—C4—H4B	109.1	C13—C14—C15	119.4 (2)
H4A—C4—H4B	107.9	C13—C14—H14	120.3
C19—C4A—C5	109.79 (16)	C15—C14—H14	120.3
C19—C4A—C4	109.90 (17)	C16—C15—C14	120.6 (2)
C5—C4A—C4	109.92 (15)	C16—C15—Cl1	119.90 (18)
C19—C4A—C8A	112.03 (15)	C14—C15—Cl1	119.51 (18)
C5—C4A—C8A	106.63 (16)	C17—C16—C15	119.8 (2)
C4—C4A—C8A	108.51 (15)	C17—C16—H16	120.1
C6—C5—C4A	112.23 (16)	C15—C16—H16	120.1
C6—C5—H5A	109.2	C16—C17—C12	121.4 (2)
C4A—C5—H5A	109.2	C16—C17—H17	119.3
C6—C5—H5B	109.2	C12—C17—H17	119.3
C4A—C5—H5B	109.2	C8—C18—H18A	109.5
H5A—C5—H5B	107.9	C8—C18—H18B	109.5
C7—C6—C5	112.20 (18)	H18A—C18—H18B	109.5
C7—C6—H6A	109.2	C8—C18—H18C	109.5
C5—C6—H6A	109.2	H18A—C18—H18C	109.5
C7—C6—H6B	109.2	H18B—C18—H18C	109.5
C5—C6—H6B	109.2	C4A—C19—H19A	109.5
H6A—C6—H6B	107.9	C4A—C19—H19B	109.5
C8—C7—C6	125.22 (19)	H19A—C19—H19B	109.5
C8—C7—H7	117.4	C4A—C19—H19C	109.5
C6—C7—H7	117.4	H19A—C19—H19C	109.5
C7—C8—C18	121.11 (19)	H19B—C19—H19C	109.5
C7—C8—C8A	120.92 (18)		
